# The Mathematics of Three N-Localizers Used Together for Stereotactic Neurosurgery

**DOI:** 10.7759/cureus.341

**Published:** 2015-10-02

**Authors:** Russell A Brown

**Affiliations:** 1 Software Development, High Technology

**Keywords:** stereotactic neurosurgery, stereotactic radiosurgery, image guidance, image-guided, computed tomography, magnetic resonance imaging, positron emission tomography (pet), n-localizer, medical imaging, brain imaging

## Abstract

The N-localizer enjoys widespread use in image-guided stereotactic neurosurgery and radiosurgery. This article derives the mathematical equations that are used with three N-localizers and provides analogies, explanations, and appendices in order to promote a deeper understanding of the mathematical principles that govern the N-localizer.

## Introduction

**Figure 1:**
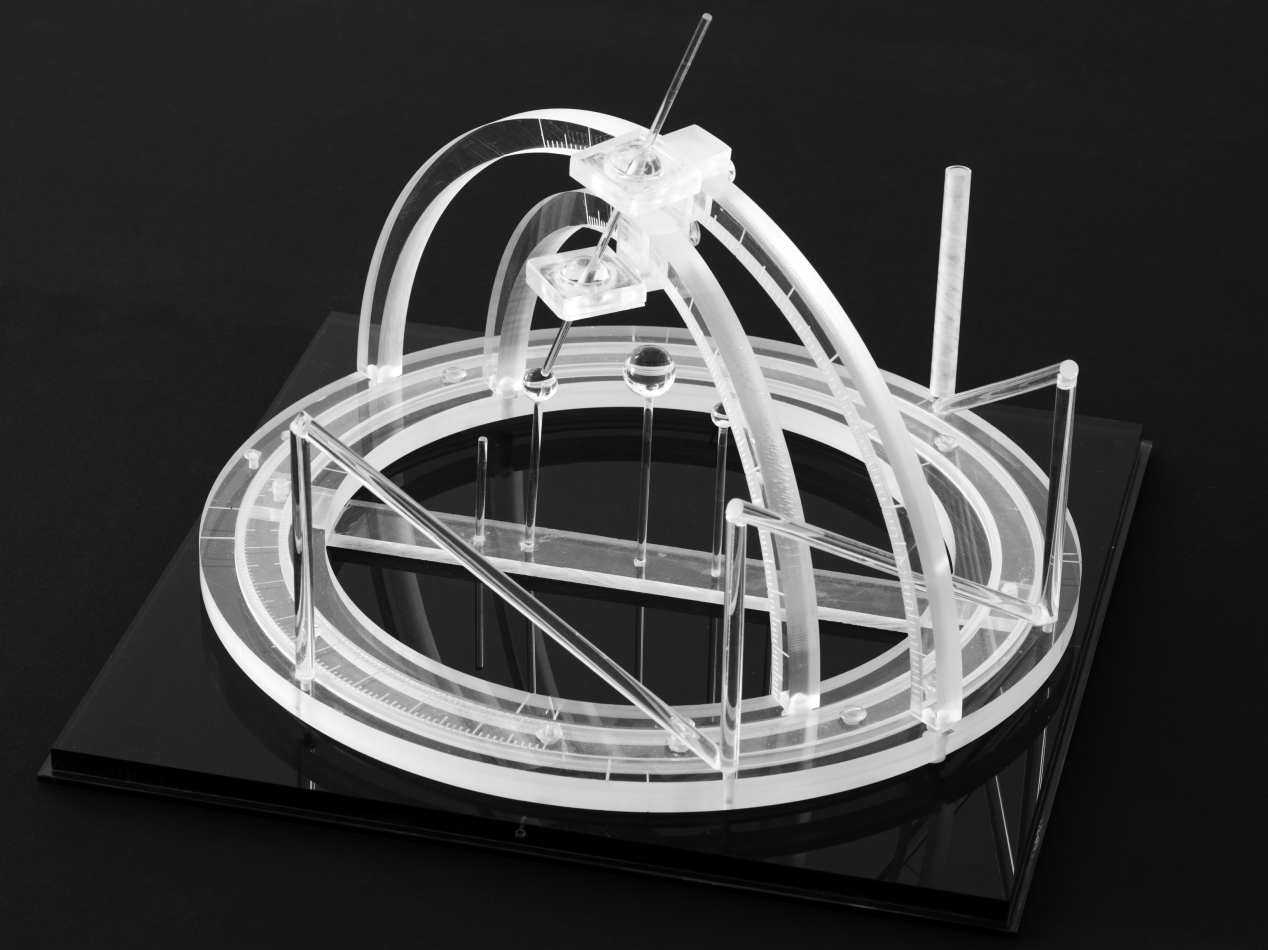
Three N-Localizers Attached to a Stereotactic Frame Three N-localizers are attached to this stereotactic frame and are merged end-to-end such that only seven rods are present. The vertical rod at the right rear of the frame is larger in diameter than the other rods. This large rod facilitates unambiguous interpretation of the fiducial circles and ellipses that the seven rods create in a tomographic image, as explained in the legend to Figure 5.

The N-localizer is a device that may be attached to a stereotactic frame (Figure 1) in order to facilitate image-guided neurosurgery and radiosurgery using tomographic images that are obtained via computed tomography (CT), magnetic resonance (MR), or positron-emission tomography (PET) [1]. The mathematics of the N-localizer have been discussed previously [2].

## Technical report

**Figure 2:**
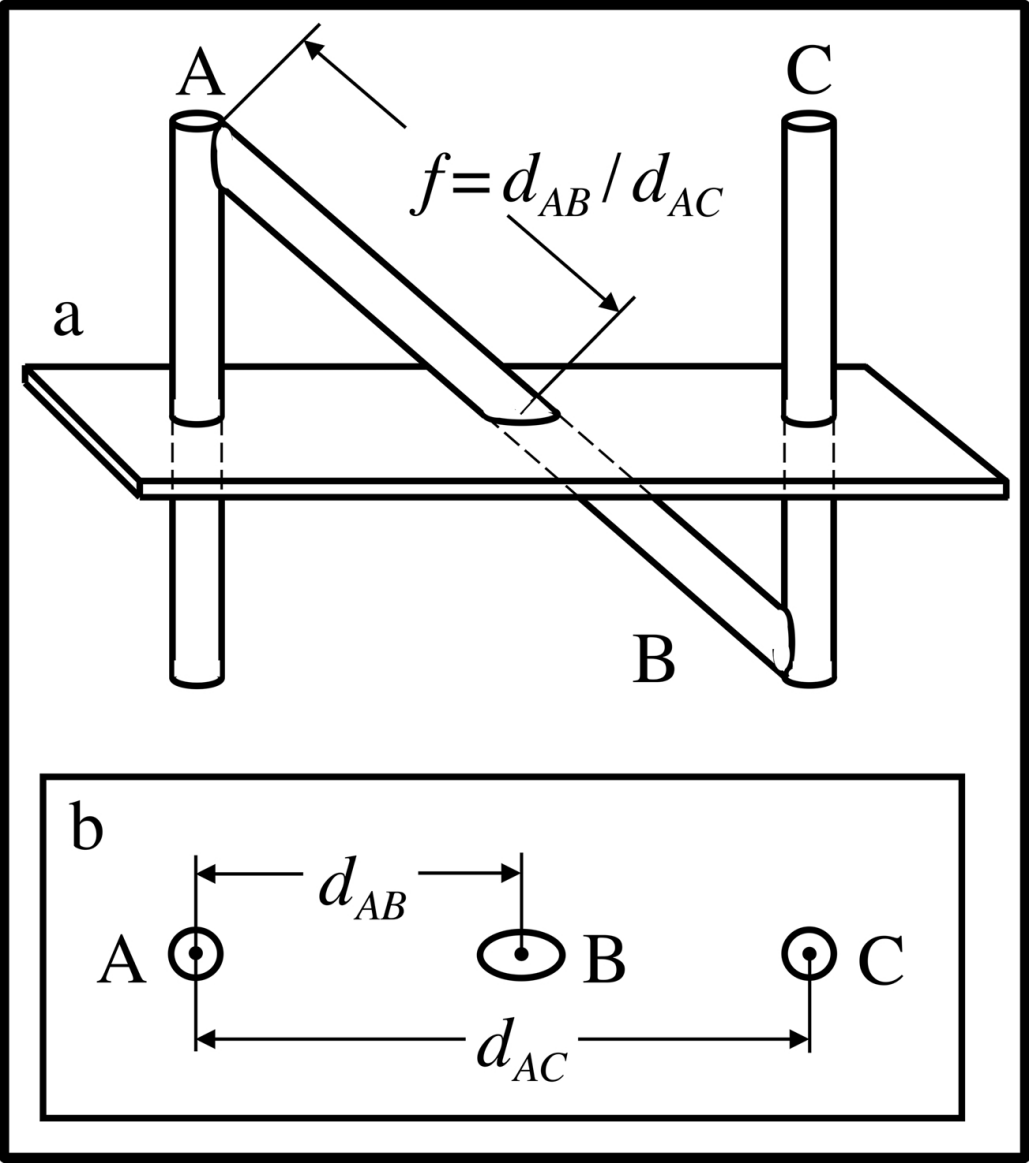
Intersection of the Tomographic Section with the N-Localizer $\mathbf{(a)}$ Side view of the N-localizer. The tomographic section intersects the rods $\mathrm A$, $\mathrm B$, and $\mathrm C$. $\mathbf{(b)}$ Tomographic image. The intersection of the tomographic section with the rods $\mathrm A$, $\mathrm B$, and $\mathrm C$ creates fiducial circles $\mathrm A$ and $\mathrm C$ and fiducial ellipse $\mathrm B$ in the tomographic image. The distance $d_{AB}$ between the centers of circle $\mathrm A$ and ellipse $\mathrm B$ and the distance $d_{AC}$ between the centers of circles $\mathrm A$ and $\mathrm C$ are used to calculate the ratio $f=d_{AB}/d_{AC}$. This ratio represents the fraction of diagonal rod $\mathrm B$ that extends from the top of rod $\mathrm A$ to the point of intersection of rod $\mathrm B$ with the tomographic section.

The N-localizer comprises a diagonal rod that extends from the top of one vertical rod to the bottom of another vertical rod (Figure 2). Assuming for the sake of simplicity that the two vertical rods are perpendicular to the tomographic section, the cross section of each vertical rod creates a fiducial circle and the cross section of the diagonal rod creates a fiducial ellipse in the tomographic image, as shown in Figure 2b. As the tomographic section moves from the top of the N-localizer towards the bottom of the N-localizer*, *i.e. towards its point of attachment to the stereotactic frame (Figure 1), the ellipse $\mathrm B$ will move away from circle $\mathrm A$ and toward circle $\mathrm C$. The relative spacing between these three fiducials permits precise localization of the tomographic section with respect to the N-localizer. The distance $d_{AB}$ between the centers of circle $\mathrm A$ and ellipse $\mathrm B$, and the distance $d_{AC}$ between the centers of circles $\mathrm A$ and $\mathrm C$ are used to calculate the ratio $f=d_{AB}/d_{AC}$. This ratio represents the fraction of diagonal rod $\mathrm B$ that extends from the top of vertical rod $\mathrm A$ to the point of intersection of rod $\mathrm B$ with the tomographic section. These linear geometric relationships exist due to the properties of similar triangles and are valid even if the vertical rods are not perpendicular to the tomographic section [3].

It is convenient to ignore the thickness of the tomographic section and to approximate the tomographic section as an infinitely thin plane. This "central" plane lies midway between the top and bottom halves of the tomographic section, analogous to the way that a slice of cheese is sandwiched between two slices of bread. The central plane approximation is susceptible to error because of the partial volume effect that derives from the several-millimeter thickness of the tomographic section [4-5]. The partial volume effect prevails because any structure that passes partially into the tomographic section, but does not span the full thickness of that section, may be visible in the tomographic image. Hence, the position of that structure is determined to only a several-millimeter error that is a well-known limitation of tomographic imaging. In the following discussion, the term "tomographic section" will be used as an abbreviation for the term "central plane of the tomographic section."

**Figure 3:**
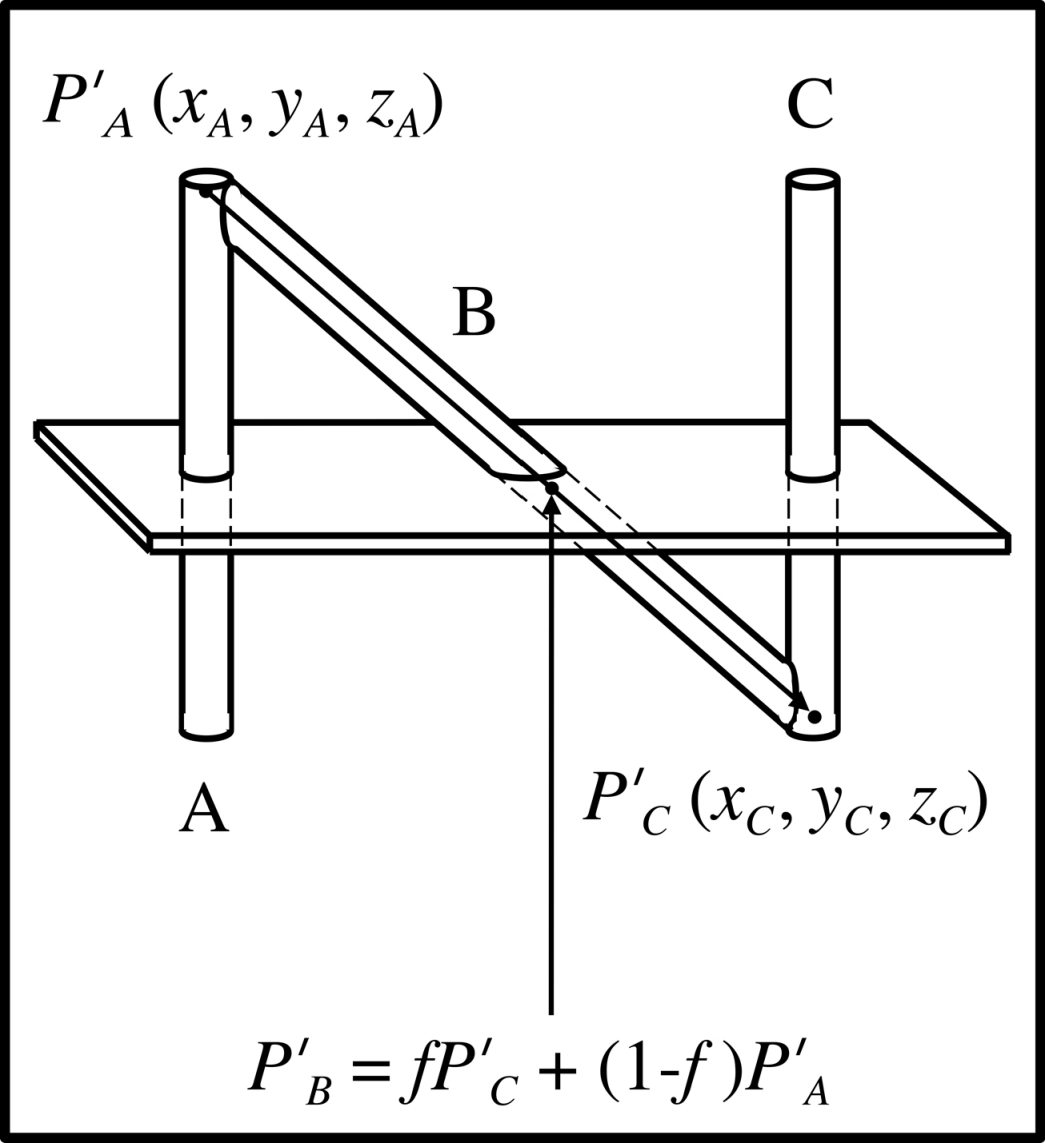
Calculation of the Point of Intersection Between the Rod B and the Tomographic Section The long axis of rod $\mathrm B$ is represented by a vector that extends from point ${P}'_{A}$ at the top of rod $\mathrm A$ to point ${P}'_{C}$ at the bottom of rod $\mathrm C$. The $\left(x_{A},y_{A},z_{A}\right)$ coordinates of point ${P}'_{A}$ and the $\left(x_{C},y_{C},z_{C}\right)$ coordinates of point ${P}'_{C}$ are known from the physical dimensions of the N-localizer. Hence, the ratio $f=d_{AB}/d_{AC}$ may be used to blend the $\left(x_{A},y_{A},z_{A}\right)$ and $\left(x_{C},y_{C},z_{C}\right)$ coordinates of points ${P}'_{A}$ and ${P}'_{C}$ via linear interpolation as indicated by Equations 1 and 2. This interpolation calculates the $\left(x_{B},y_{B},z_{B}\right)$ coordinates of the point of intersection ${P}'_{B}$ between the long axis of rod $\mathrm B$ and the tomographic section.

The fraction $f$ is used to calculate the $\left(x,y,z\right)$ coordinates of the point of intersection ${P}'_{B}$ between the long axis of rod $\mathrm B$ and the tomographic section (Figure 3). In this figure, points ${P}'_{A}$ and ${P}'_{C}$ represent the beginning and end, respectively, of the vector that extends from the top of rod $\mathrm A$ to the bottom of rod $\mathrm C$. This vector coincides with the long axis of rod $\mathrm B$. The $\left(x_{A},y_{A},z_{A}\right)$ coordinates of the beginning point ${P}'_{A}$ and the $\left(x_{C},y_{C},z_{C}\right)$ coordinates of the end point ${P}'_{C}$ are known from the physical dimensions of the N-localizer. Hence, linear interpolation may be used to blend points ${P}'_{A}$ and ${P}'_{C}$ to obtain the $\left(x_{B},y_{B},z_{B}\right)$ coordinates of the point of intersection ${P}'_{B}$ between the long axis of rod $\mathrm B$ and the tomographic section

$${P}'_{B}={P}'_{A}+f\left({P}'_{C}-{P}'_{A}\right)=f{P}'_{C}+\left(1-f\right){P}'_{A}\;\;\;\;\;\;\;\;\;\;\left(1\right)$$

The vector form of Equation 1 shows explicitly the $\left(x,y,z\right)$ coordinates of points ${P}'_{A}$, ${P}'_{B}$, and ${P}'_{C}$

$$\begin{bmatrix}x_{B} \; y_{B} \; z_{B}\end{bmatrix} = f \begin{bmatrix}x_{C} \; y_{C} \; z_{C}\end{bmatrix} +\left(1-f \right) \begin{bmatrix}x_{A} \; y_{A} \; z_{A}\end{bmatrix}\;\;\;\;\;\;\;\;\;\;\left(2\right)$$

Equation 1 or 2 may be used to calculate the $\left(x_{B},y_{B},z_{B}\right)$ coordinates of the point of intersection ${P}'_{B}$ between the long axis of rod $\mathrm B$ and the tomographic section. The point ${P}'_{B}$, which lies on the long axis of rod $\mathrm B$ in the three-dimensional coordinate system of the N-localizer, corresponds to the analogous point $P_{B}$, which lies at the center of ellipse $\mathrm B$ in the two-dimensional coordinate system of the tomographic image (Figure 2b). Hence, there is a one-to-one linear mapping between a point from the N-localizer and a point from the tomographic image.

**Figure 4:**
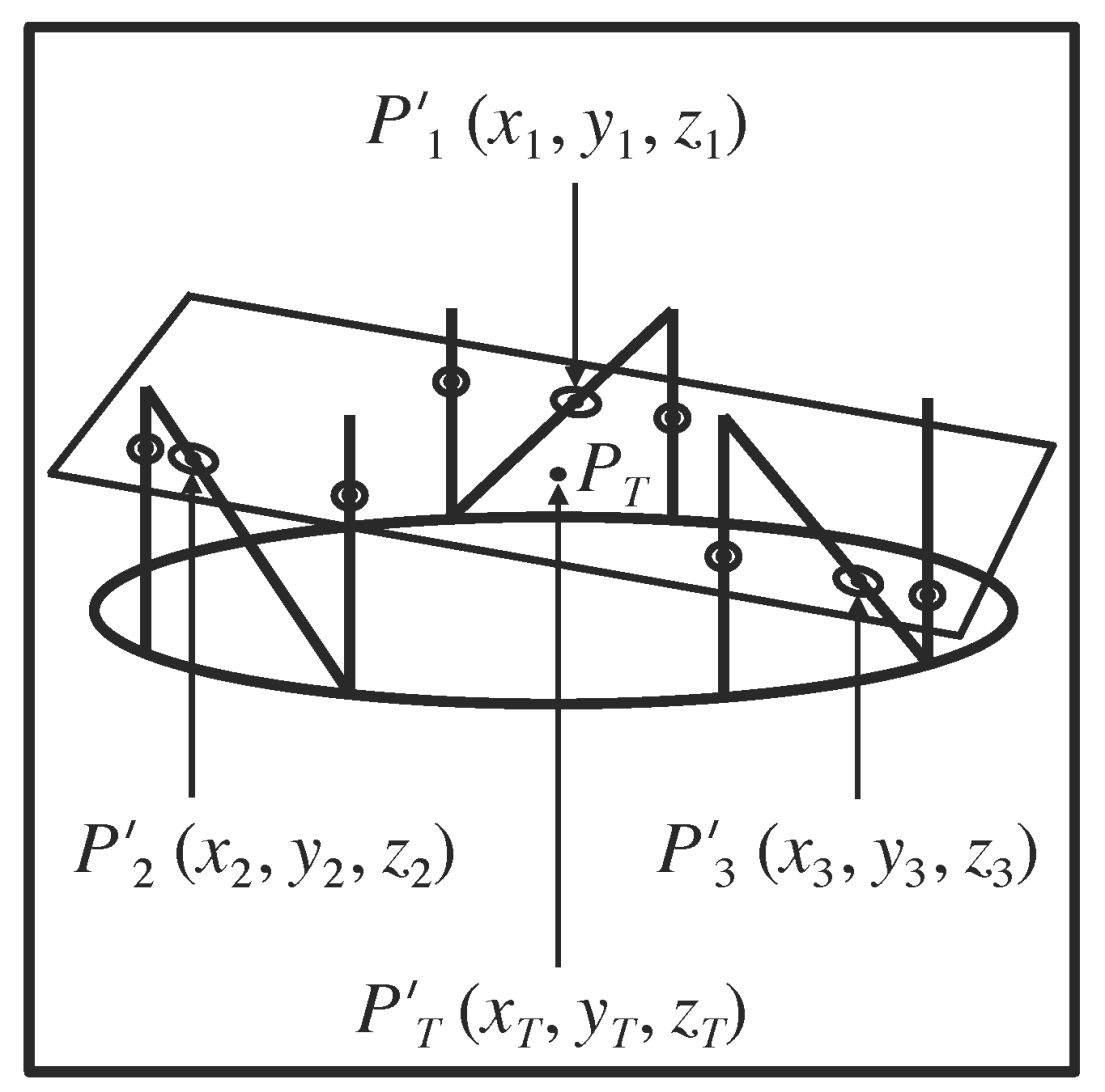
Representation of the Tomographic Section in the Three-Dimensional Coordinate System of the Stereotactic Frame The quadrilateral represents the tomographic section. The large oval depicts the circular base of the stereotactic frame (in perspective). The vertical and diagonal lines that are attached to the large oval represent the nine rods. The centers of the six fiducial circles and the three fiducial ellipses that are created in the tomographic image by these nine rods are shown as points that lie in the tomographic section. The tomographic section intersects the long axes of the three diagonal rods at the points ${P}'_{1}$, ${P}'_{2}$, and ${P}'_{3}$ that coincide with the respective centers $P_{1}$, $P_{2}$, and $P_{3}$ of the three ellipses (Figure 6). The $\left(x_{1},y_{1},z_{1}\right)$, $\left(x_{2},y_{2},z_{2}\right)$, and $\left(x_{3},y_{3},z_{3}\right)$ coordinates of the respective points of intersection ${P}'_{1}$, ${P}'_{2}$, and ${P}'_{3}$ are calculated in the three-dimensional coordinate system of the stereotactic frame using Equations 1 and 2. Because these three points determine the spatial orientation of a plane in three-dimensional space, the spatial orientation of the tomographic section is determined with respect to the stereotactic frame. The target point ${P}'_{T}$ lies in the tomographic section. The $\left(x_{T},y_{T},z_{T}\right)$ coordinates of this target point are calculated in the three-dimensional coordinate system of the stereotactic frame using Equation 6.

The attachment of three N-localizers to a stereotactic frame permits calculation of the $\left(x_{B_{1}},y_{B_{1}},z_{B_{1}}\right)$, $\left(x_{B_{2}},y_{B_{2}},z_{B_{2}}\right)$, and $\left(x_{B_{3}},y_{B_{3}},z_{B_{3}}\right)$ coordinates for the three respective points, ${P}'_{B_{1}}$, ${P}'_{B_{2}}$, and ${P}'_{B_{3}}$, in the three-dimensional coordinate system of the stereotactic frame (Figure 4). These three points correspond respectively to the three analogous points, $P_{B_{1}}$, $P_{B_{2}}$, and $P_{B_{3}}$, in the two-dimensional coordinate system of the tomographic image. In the following discussion, the symbols, ${P}'_{1}$, ${P}'_{2}$, and ${P}'_{3}$, will be used as a shorthand notation for ${P}'_{B_{1}}$, ${P}'_{B_{2}}$, and ${P}'_{B_{3}}$. The symbols, $P_{1}$, $P_{2}$, and $P_{3}$, will be used as a shorthand notation for $P_{B_{1}}$, $P_{B_{2}}$, and $P_{B_{3}}$.

**Figure 5:**
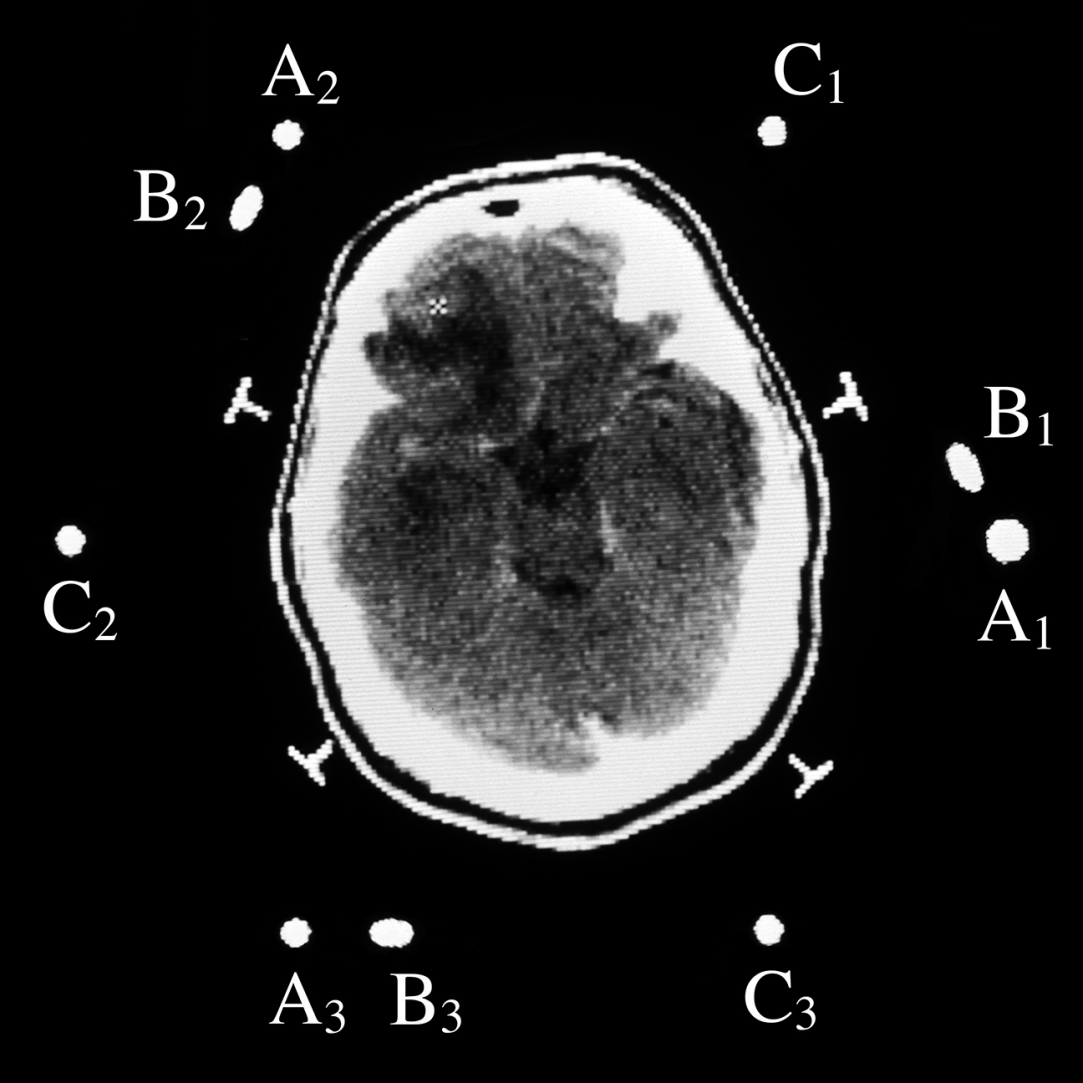
CT Image with Three Sets of Fiducials CT image of a patient to whom a BRW CT localizer frame (Integra Radionics Inc., Burlington, MA), which comprises three N-localizers, is attached. The cross sections of the three N-localizers create three sets of fiducials $\left\{ \mathrm{A_1,B_1,C_1} \right\}$, $\left\{ \mathrm{A_2,B_2,C_2} \right\}$, and $\left\{ \mathrm{A_3,B_3,C_3} \right\}$ in the CT image. The cursor indicates the target point $P_T$. The large vertical rod $\mathrm{A_1}$ allows it to be unambiguously distinguished from the other vertical rods and provides a visual cue that this figure is rotated approximately 90 degrees clockwise relative to Figure 6 [6].

**Figure 6:**
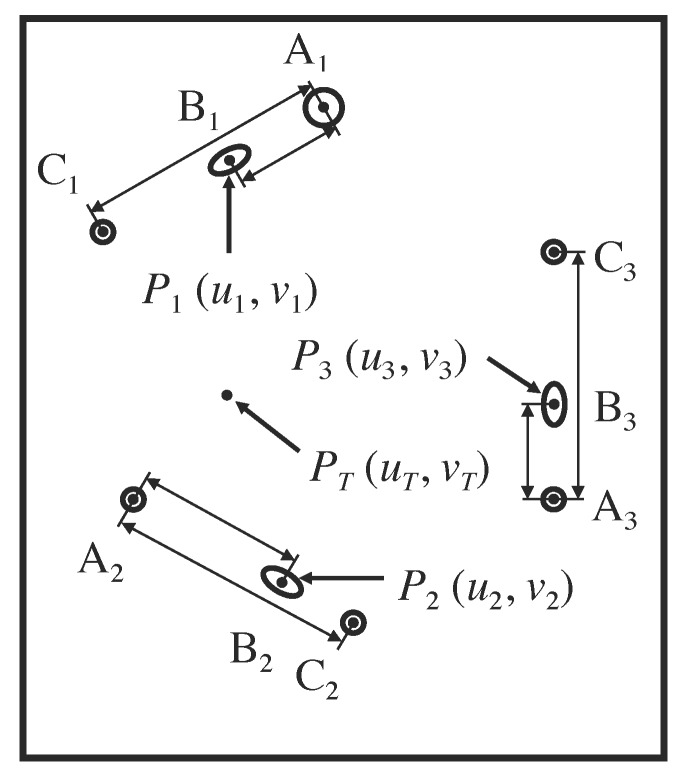
Representation of the Two-Dimensional Coordinate System of the Tomographic Image The cross sections of the three N-localizers create three sets of fiducials $\left \{ \mathrm{A_1,B_1,C_1} \right \}$, $\left \{ \mathrm{A_2,B_2,C_2} \right \}$, and $\left \{ \mathrm{A_3,B_3,C_3} \right \}$ in the tomographic image. Each set contains two circles and one ellipse that are collinear. For each set, the short double-ended arrows indicate the distance $d_{AB}$ between the centers of circle $\mathrm A$ and ellipse $\mathrm B$ and the long double-ended arrows indicate the distance $d_{AC}$ between the centers of circles $\mathrm A$ and $\mathrm C$. The centers $P_{1}$, $P_{2}$, and $P_{3}$ of the three ellipses coincide with the respective points of intersection ${P}'_{1}$, ${P}'_{2}$, and ${P}'_{3}$ of the long axes of the three diagonal rods with the tomographic section (Figure 4). The $\left(u_{1},v_{1}\right)$, $\left(u_{2},v_{2}\right)$, and $\left(u_{3},v_{3}\right)$ coordinates of the centers $P_{1}$, $P_{2}$, and $P_{3}$ correspond respectively to the $\left(x_{1},y_{1},z_{1}\right)$, $\left(x_{2},y_{2},z_{2}\right)$, and $\left(x_{3},y_{3},z_{3}\right)$ coordinates of the points of intersection ${P}'_{1}$, ${P}'_{2}$, and ${P}'_{3}$. The target point $P_{T}$ has $\left(u_{T},v_{T}\right)$ coordinates in the two-dimensional coordinate system of the tomographic image. The $\left(x_{T},y_{T},z_{T}\right)$ coordinates of the analogous target point ${P}'_{T}$ are calculated in the three-dimensional coordinate system of the stereotactic frame using Equation 6.

The three points, ${P}'_{1}$, ${P}'_{2}$, and ${P}'_{3}$, lie on the three respective diagonal rods, $\mathrm B_1$, $\mathrm B_2$, and $\mathrm B_3$, and have respective $\left(x,y,z\right)$ coordinates, $\left(x_{1},y_{1},z_{1}\right)$, $\left(x_{2},y_{2},z_{2}\right)$, and $\left(x_{3},y_{3},z_{3}\right)$, in the three-dimensional coordinate system of the stereotactic frame (Figure 4). The analogous three points, $P_{1}$, $P_{2}$, and $P_{3}$, lie at the centers of the three respective ellipses, $\mathrm B_1$, $\mathrm B_2$, and $\mathrm B_3$, and have $\left(u,v\right)$ coordinates, $\left(u_{1},v_{1}\right)$, $\left(u_{2},v_{2}\right)$, and $\left(u_{3},v_{3}\right)$, in the two-dimensional coordinate system of the tomographic image (Figures 5-6).

In order to facilitate calculation of the $\left(x_{T},y_{T},z_{T}\right)$ coordinates of the target point ${P}'_{T}$, it is convenient to project the $\left(u_{1},v_{1}\right)$, $\left(u_{2},v_{2}\right)$, and $\left(u_{3},v_{3}\right)$ coordinates of the three centers, $P_{1}$, $P_{2}$, and $P_{3}$, of the ellipses onto the $w = 1$ plane in three-dimensional space by appending a third coordinate $w = 1$ to create $\left(u_{1},v_{1},1\right)$, $\left(u_{2},v_{2},1\right)$, and $\left(u_{3},v_{3},1\right)$ coordinates. The $w$-coordinate may be set arbitrarily to any non-zero value, e.g., 1, so long as same value of $w$ is used for each of the three $w$-coordinates. The equations that are presented in the remainder of this article assume that a value of $w = 1$ has been used to project the $\left(u_{1},v_{1}\right)$, $\left(u_{2},v_{2}\right)$, and $\left(u_{3},v_{3}\right)$ coordinates. If a value of $w \neq 1$ were used instead of $w = 1$ to project these coordinates, the equations that are presented in the remainder of this article would no longer apply and would require revision so that the calculations that these equations describe may produce correct results.

Because three points determine the orientation of a plane in three-dimensional space, the three coordinates, $\left(x_{1},y_{1},z_{1}\right)$, $\left(x_{2},y_{2},z_{2}\right)$, and $\left(x_{3},y_{3},z_{3}\right)$, together with the three coordinates, $\left(u_{1},v_{1}\right)$, $\left(u_{2},v_{2}\right)$, and $\left(u_{3},v_{3}\right)$, determine the spatial orientation of the tomographic section with respect to the stereotactic frame. This spatial orientation or linear mapping is specified by the matrix elements $m_{11}$ through $m_{33}$ in the matrix equation

$$\begin{bmatrix} x_1 & y_1 & z_1 \\ x_2 & y_2 & z_2 \\ x_3 & y_3 & z_3 \end{bmatrix} = \begin{bmatrix} u_1 & v_1 & 1 \\ u_2 & v_2 & 1 \\ u_3 & v_3 & 1 \end{bmatrix} \begin{bmatrix} m_{11} & m_{12} & m_{13} \\ m_{21} & m_{22} & m_{23} \\ m_{31} & m_{32} & m_{33} \end{bmatrix}\;\;\;\;\;\;\;\;\;\;\left(3\right)$$

Equation 3 represents concisely a system of nine simultaneous linear equations that determine the spatial orientation of the tomographic section with respect to the stereotactic frame. This equation transforms the $\left(u_{1},v_{1}\right)$, $\left(u_{2},v_{2}\right)$, and $\left(u_{3},v_{3}\right)$ coordinates from the two-dimensional coordinate system of the tomographic image to create $\left(x_{1},y_{1},z_{1}\right)$, $\left(x_{2},y_{2},z_{2}\right)$, and $\left(x_{3},y_{3},z_{3}\right)$ coordinates in the three-dimensional coordinate system of the stereotactic frame.

Equation 3 assumes a linear mapping from the two-dimensional coordinate system of the tomographic image to the three-dimensional coordinate system of the stereotactic frame. Magnetic resonance (MR) images are susceptible to nonlinear distortion that invalidates this linear mapping and nullifies the applicability of Equation 3. For this reason, the Brown-Roberts-Wells (BRW) stereotactic frame [7] that was used initially with computed tomography (CT) required modification to eliminate nonlinear distortion of MR images. The CT-compatible BRW frame comprised an aluminum ring in which the magnetic field that the MR scanner generated to acquire MR images induced eddy currents. Those eddy currents distorted the MR images. Replacing one section of the aluminum ring with a nonmetallic insert prevented magnetically induced circumferential eddy currents and eliminated nonlinear distortion of the MR images [8].

An analogy provides insight into how the transformation of Equation 3 operates. Consider the tomographic image to be an elastic membrane. The transformation describes the process of stretching the membrane in the plane of the tomographic image, rotating the membrane about an axis that is normal to the plane of the tomographic image, tilting the membrane, if necessary, so that it is not parallel to the base of the stereotactic frame, and lifting the membrane into place upon the scaffold of the three N-localizers, such that the three points, $P_{1}$, $P_{2}$, and $P_{3}$, from the tomographic image precisely coincide with the respective three points, ${P}'_{1}$, ${P}'_{2}$, and ${P}'_{3}$, from the stereotactic frame. Then, any other point that lies on the membrane, e.g., the target point $P_{T}$, is transformed by the same stretching, rotating, tilting, and lifting processes that transformed the three points, $P_{1}$, $P_{2}$, and $P_{3}$. In this manner, the $\left(u_{T},v_{T}\right)$ coordinates of the target point $P_{T}$ may be transformed from the two-dimensional coordinate system of the tomographic image into the three-dimensional coordinate system of the stereotactic frame to produce the $\left(x_{T},y_{T},z_{T}\right)$ coordinates of the analogous target point ${P}'_{T}$.

Equation 3 may be rewritten in more compact form as

$$\mathrm{\textbf{F}}=\mathrm{\textbf{SM}}\;\;\;\;\;\;\;\;\;\;\left(4 \right )$$

In Equation 4, $\mathrm{\textbf{F}}$ represents the matrix of $\left(x_{1},y_{1},z_{1}\right)$, $\left(x_{2},y_{2},z_{2}\right)$, and $\left(x_{3},y_{3},z_{3}\right)$ coordinates in the coordinate system of the stereotactic frame. $\mathrm{\textbf{S}}$ represents the matrix of $\left(u_{1},v_{1},1\right)$, $\left(u_{2},v_{2},1\right)$, and $\left(u_{3},v_{3},1\right)$ coordinates in the coordinate system of the tomographic image. $\mathrm{\textbf{M}}$ represents the matrix of elements, $m_{11}$ through $m_{33}$, that defines the transformation from the two-dimensional coordinate system of the tomographic image to the three-dimensional coordinate system of the stereotactic frame.

The elements of $\mathrm{\textbf{F}}$ and $\mathrm{\textbf{S}}$ are known, but the elements of $\mathrm{\textbf{M}}$ are unknown. It is possible to solve Equation 4 for the elements of $\mathrm{\textbf{M}}$

$$\mathrm{\textbf{M}}=\mathrm{\textbf{S}}^{-1}\mathrm{\textbf{F}}\;\;\;\;\;\;\;\;\;\;\left(5\right)$$

In this equation, $\mathrm{\textbf{S}}^{-1}$ represents the inverse of matrix $\mathrm{\textbf{S}}$. The inverse of $\mathrm{\textbf{S}}$ is guaranteed to exist so long as the $\left(u_{1},v_{1},1\right)$, $\left(u_{2},v_{2},1\right)$, and $\left(u_{3},v_{3},1\right)$ coordinates of the centers of the three ellipses $\mathrm{B}_1$, $\mathrm{B}_2$, and $\mathrm{B}_3$ are not collinear. This non-collinearity is enforced by careful design of the stereotactic frame, as will be explained below in the Discussion.

Once the elements of matrix $\mathrm{\textbf{M}}$ have been calculated via Equation 5, it is possible to transform the $\left(u_{T},v_{T}\right)$ coordinates of the target point $P_{T}$ from the two-dimensional coordinate system of the tomographic image to the three dimensional coordinate system of the stereotactic frame to obtain the $\left(x_{T},y_{T},z_{T}\right)$ coordinates of the analogous target point ${P}'_{T}$. In order to accomplish this transformation, the $\left(u_{T},v_{T}\right)$ coordinates of $P_{T}$ are used to form the vector $\left [ u_{T}\; v_{T}\; 1 \right ]$ that is post-multiplied by matrix $\mathrm{\textbf{M}}$ to obtain the vector $\left [ x_{T}\; y_{T}\; z_{T}\right ]$ that contains the $\left(x_{T},y_{T},z_{T}\right)$ coordinates of ${P}'_{T}$

$$\left [ x_{T}\; y_{T}\; z_{T}\right ] = \left [ u_{T}\; v_{T}\; 1 \right ] \mathrm{\textbf{M}}\;\;\;\;\;\;\;\;\;\;\left(6\right)$$

Moreover, it is possible to calculate the inverse of matrix $\mathrm{\textbf{M}}$

$$\mathrm{\textbf{M}}^{-1}=\mathrm{\textbf{F}}^{-1}\mathrm{\textbf{S}}\;\;\;\;\;\;\;\;\;\;\left(7\right)$$

The inverse matrix $\mathrm{\textbf{M}}^{-1}$ may be used perform a transformation analogous to the transformation of Equation 6 but in the reverse direction. This reverse transformation transforms the $\left(x_{Q},y_{Q},z_{Q}\right)$ coordinates of a point ${P}'_Q$ from the three-dimensional coordinate system of the stereotactic frame to the two-dimensional coordinate system of the tomographic image to obtain the $\left(u_Q, v_Q\right)$ coordinates of the analogous point $P_Q$. In order to accomplish this reverse transformation, the $\left(x_{Q},y_{Q},z_{Q}\right)$ coordinates of ${P}'_Q$ are used to form the vector $\left [ x_{Q}\; y_{Q}\; z_{Q}\right ]$ that is post-multiplied by matrix $\mathrm{\textbf{M}}^{-1}$ to obtain the vector $\left [ u_{Q}\; v_{Q}\; w_{Q}\right ]$ that contains the $\left(u_{Q},v_{Q},w_{Q}\right)$ coordinates of $P_Q$

$$\left [ u_{Q}\; v_{Q}\; w_{Q}\right ] = \left [ x_{Q}\; y_{Q}\; z_{Q}\right ] \mathrm{\textbf{M}}^{-1}\;\;\;\;\;\;\;\;\;\;\left(8\right)$$

Equation 8 yields $\left(u_{Q},v_{Q},w_{Q}\right)$ coordinates for $P_Q$ instead of $\left(u_{Q},v_{Q},1\right)$ coordinates. The $w$-coordinate $w_Q$ equals 1 if and only if the point ${P}'_Q$ lies in the tomographic section that corresponds to the $w=1$ plane in three-dimensional space [2]. Similarly, $P_Q$ appears in the two-dimensional tomographic image if and only if $w_Q=1$. In the case that ${P}'_Q$ does not lie in the tomographic section, $w_Q \neq 1$ so $P_Q$ does not appear in the tomographic image.

**Figure 7:**
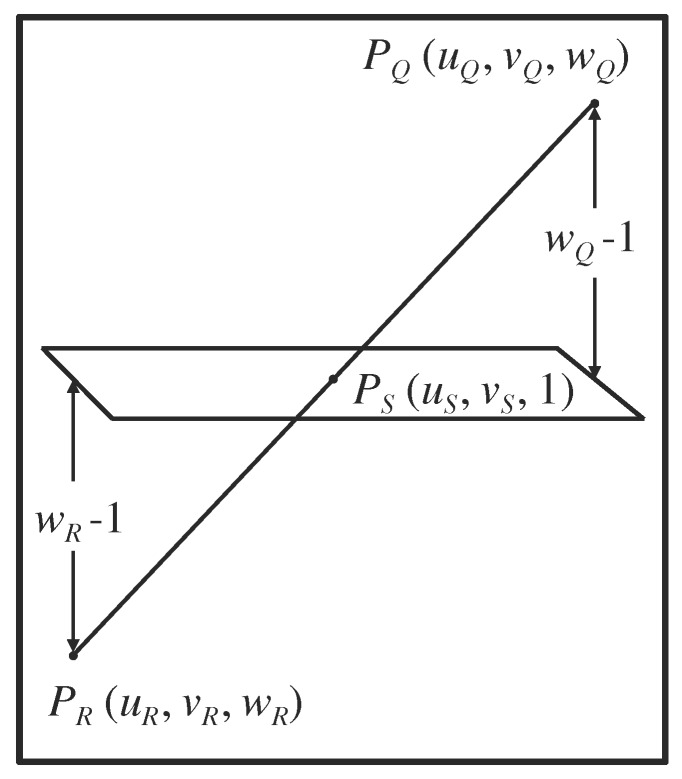
Interpolation Within the Vector from $P_Q$ to $P_R$ in Order to Obtain the Point $P_S$ that Appears in the Tomographic Image The points $P_Q$ and $P_R$ are located on opposite sides of an intermediate tomographic image for which $w_S=1$. The distances $w_Q-1$ and $w_Q-w_R=\left(w_Q-1\right)-\left(w_R-1\right)$ are used to obtain the interpolant$$t=\frac{w_Q-1}{w_Q-w_R}$$This interpolant is used to calculate the $\left(u_S,v_S\right)$ coordinates of the point $P_S$ that appears in the tomographic image.

One case where $P_Q$ does not appear in the tomographic image occurs when the point ${P}'_Q$ and a second point ${P}'_R$ define the intended trajectory of a surgical probe but neither ${P}'_Q$ nor ${P}'_R$ lies in an intermediate tomographic section (Figure 7). In this case, $w_Q \neq 1$ and $w_R \neq 1$, so neither $P_Q$ nor $P_R$ appears in the intermediate tomographic image.

**Figure 8:**
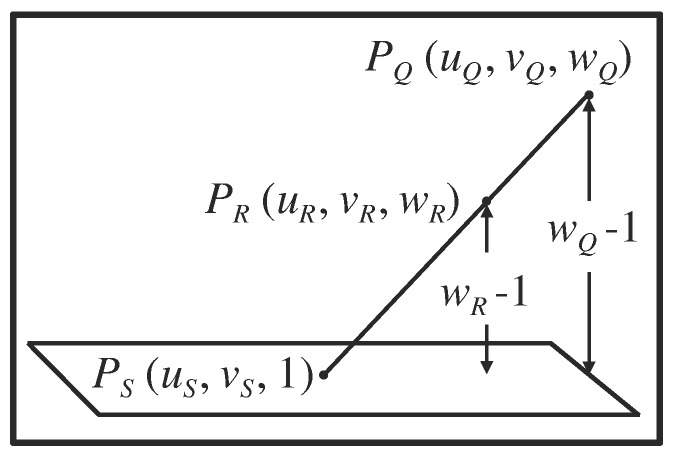
Extrapolation Beyond the Vector from $P_Q$ to $P_R$ in Order to Obtain the Point $P_S$ that Appears in the Tomographic Image The points $P_Q$ and $P_R$ are located on the same side of a non-intermediate tomographic image for which $w_S=1$. The distances $w_Q-1$ and $w_Q-w_R=\left(w_Q-1\right)-\left(w_R-1\right)$ are used to obtain the extrapolant$$t=\frac{w_Q-1}{w_Q-w_R}$$This extrapolant is used to calculate the $\left(u_S,v_S\right)$ coordinates of the point $P_S$ that appears in the tomographic image.

However, in this case, the neurosurgeon may wish to know where the intended probe trajectory would intersect the intermediate tomographic section. In order to provide this information, the points $P_Q$ and $P_R$ are used to define the vector from $P_Q$ to $P_R$. This vector is then used to calculate the $\left(u_S,v_S\right)$ coordinates of a third point $P_S$ for which $w_S=1$ (Figure 7). Because $w_S=1$, $P_S$ appears in the intermediate tomographic image; hence, a mark may be superimposed on that tomographic image at the $\left(u_S,v_S\right)$ coordinates $P_S$ to indicate where the intended probe trajectory would intersect the intermediate tomographic section [2]. It is possible to distinguish two configurations of $P_Q$ and $P_R$ relative to an intermediate tomographic image: $w_Q > 1 > w_R$ and $w_Q > w_R > 1$. All other configurations can be made to conform to one of these two configurations via interchange of $P_Q$ and $P_R$ and/or inverting the signs of both $w_Q$ and $w_R$. The configuration $w_Q > 1 > w_R$ specifies that $P_Q$ and $P_R$ are located on opposite sides of an intermediate tomographic image; thus, linear interpolation may be used to calculate $P_S$ (Figure 7). The configuration $w_Q > w_R > 1$ specifies that $P_Q$ and $P_R$ are located on the same side of a non-intermediate tomographic image; thus, linear extrapolation may be used to calculate $P_S$ (Figure 8).

For either interpolation or extrapolation, the term

$$t=\frac{w_Q-1}{w_Q-w_R}\;\;\;\;\;\;\;\;\;\;\left(9 \right )$$

is used to calculate the $\left(u_S,v_S\right)$ coordinates of $P_S$ by blending the $\left(u_Q,v_Q\right)$ and $\left(u_R,v_R\right)$ coordinates of $P_Q$ and $P_R$

$$P_S=P_Q+t\left(P_R-P_Q\right)=t\,P_R+\left(1-t\right)P_Q\;\;\;\;\;\;\;\;\;\;\left(10 \right )$$

The vector form of Equation 10 shows explicitly the $\left(u_{Q},v_{Q},w_{Q}\right)$, $\left(u_{R},v_{R},w_{R}\right)$, and $\left(u_{S},v_{S},w_{S}\right)$ coordinates of the respective points $P_{Q}$, $P_{R}$ and $P_{S}$

$$\begin{bmatrix} u_{S} \; v_{S} \; w_{S} \end{bmatrix} = t \begin{bmatrix} u_{R} \; v_{R} \; w_{R} \end{bmatrix} +\left(1-t \right) \begin{bmatrix} u_{Q} \; v_{Q} \; w_{Q} \end{bmatrix}\;\;\;\;\;\;\;\;\;\;\left(11\right)$$

It is necessary to calculate only the $\left(u_S,v_S\right)$ coordinates of $P_S$ using Equation 11 because $w_S=1$ due to the definition of $t$ in Equation 9. It is possible to prove that $w_S=1$ by substituting Equation 9 into Equation 11 then expanding the resulting expression in the $w$-coordinate to obtain

$$w_S=\left(\frac{w_Q-1}{w_Q-w_R}\right)w_R+\left(1-\frac{w_Q-1}{w_Q-w_R}\right)w_Q=\frac{\left(w_Q-1\right)w_R+\left(1-w_R\right)w_Q}{{w_Q-w_R}}=1\;\;\;\;\;\left(12 \right )$$

## Discussion

The above mathematical formulation imposes some constraints on the physical design of the stereotactic frame and on the mathematical model of that frame. Specifically, Equations 5 and 7 require that the mathematical model of the frame permit the inverse matrices, $\mathbf{S}^{-1}$ and $\mathbf{F}^{-1}$, to exist.

The inverse matrix $\mathbf{S}^{-1}$ exists if and only if the points $P_{1}$, $P_{2}$, and $P_{3}$ are neither collinear nor lie on a plane that passes through the origin of the $\left(u, v, w\right)$ coordinate system. Similarly, the inverse $\mathbf{F}^{-1}$ exists if and only if the points ${P}'_{1}$, ${P}'_{2}$, and ${P}'_{3}$ are neither collinear nor lie on a plane that passes through the origin of the $\left(x, y, z\right)$ coordinate system.

The collinearity requirement is satisfied for both $\mathbf{F}^{-1}$ and $\mathbf{S}^{-1}$ by judiciously choosing the positions of the three N-localizers relative to the stereotactic frame. Because for contemporary stereotactic frames the N-localizers are positioned either around the circumference of a circle or on the faces of a cube, neither the points $P_{1}$, $P_{2}$, and $P_{3}$ nor the points ${P}'_{1}$, ${P}'_{2}$, and ${P}'_{3}$ can possibly be collinear.

The requirement that the points $P_{1}$, $P_{2}$, and $P_{3}$ do not lie on a plane that passes through the origin of the $\left(u, v, w\right)$ coordinate system is satisfied by choosing $w=1$ to project the $\left(u_{1},v_{1}\right)$, $\left(u_{2},v_{2}\right)$, and $\left(u_{3},v_{3}\right)$ coordinates to create $\left(u_{1},v_{1},1\right)$, $\left(u_{2},v_{2},1\right)$, and $\left(u_{3},v_{3},1\right)$ coordinates.

The requirement that the points ${P}'_{1}$, ${P}'_{2}$, and ${P}'_{3}$ do not lie on a plane that passes through the origin of the $\left(x, y, z\right)$ coordinate system may be satisfied by judiciously defining the $\left(x, y, z\right)$ coordinate system of the stereotactic frame, such that the $z$-coordinate cannot equal zero anywhere along the diagonal rods. Figure 3 demonstrates that Equations 1 and 2 will never produce $z=0$ along the diagonal rods so long as the interval from $z_A$ to $z_C$ does not contain zero. One way to satisfy this requirement is to define the origin of the $\left(x, y, z\right)$ coordinate system of the stereotactic frame to lie below the base of the N-localizers, such that the $z$-coordinate of the origin is always less than the $z_C$-coordinate of point $P_C$.

When all three of the above requirements are satisfied, the matrix $\mathbf{M}$ will correctly transform the $\left(u_{1},v_{1},1\right)$, $\left(u_{2},v_{2},1\right)$, and $\left(u_{3},v_{3},1\right)$ coordinates of points $P_{1}$, $P_{2}$, and $P_{3}$ from the two-dimensional coordinate system of the tomographic image to create the $\left(x_{1},y_{1},z_{1}\right)$, $\left(x_{2},y_{2},z_{2}\right)$, and $\left(x_{3},y_{3},z_{3}\right)$ coordinates of points ${P}'_{1}$, ${P}'_{2}$, and ${P}'_{3}$ in the three-dimensional coordinate system of the stereotactic frame as indicated by Equation 4. Also, the inverse matrix $\mathbf{M}^{-1}$ will correctly perform the inverse of that transformation.

## Conclusions

The N-localizer is a simple yet powerful tool for image-guided stereotactic neurosurgery and radiosurgery. The N-localizer enables the transformation of $\left ( u,v \right )$ coordinates from the two-dimensional coordinate system of the computed tomography (CT), magnetic resonance (MR) or positron-emission tomography (PET) image to the three-dimensional coordinate system of the stereotactic frame to obtain $\left ( x,y,z \right )$ coordinates. The matrix that accomplishes this transformation may be inverted; the resulting inverse matrix enables the transformation of $\left ( x,y,z \right )$ coordinates from the three-dimensional coordinate system of the stereotactic frame to the two-dimensional coordinate system of the computed tomography or magnetic resonance image to obtain $\left(u,v\right)$ coordinates.

## Figures and Tables

**Figure 9:**
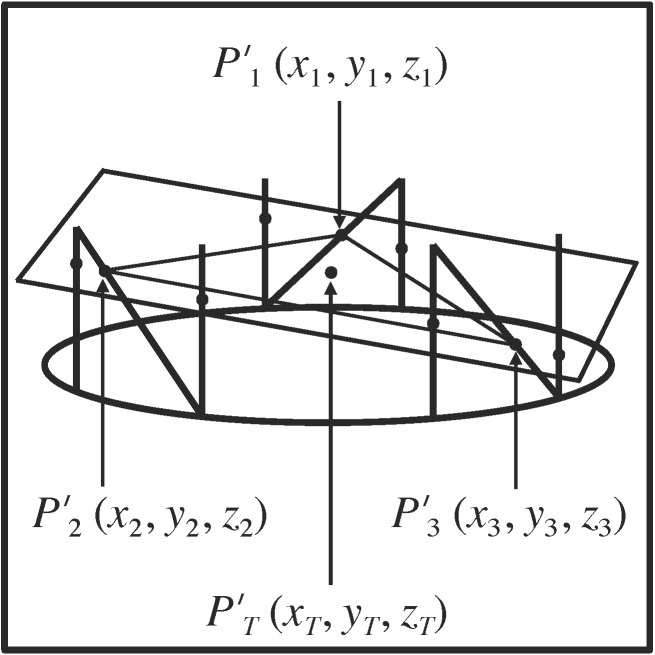
Representation of the Tomographic Section in the Three-Dimensional Coordinate System of the Stereotactic Frame The quadrilateral represents the tomographic section. The large oval depicts the circular base of the stereotactic frame (in perspective). The vertical and diagonal lines that are attached to the large oval represent the nine rods. The centers of the six fiducial circles and the three fiducial ellipses that are created in the tomographic image by these nine rods are shown as points that lie in the tomographic section. The tomographic section intersects the long axes of the three diagonal rods at points ${P}'_{1}$, ${P}'_{2}$, and ${P}'_{3}$ that coincide with the respective centers $P_{1}$, $P_{2}$, and $P_{3}$ of the three ellipses (Figure 10). The target point ${P}'_{T}$ lies in the plane of the triangle ${P}'_{1}{P}'_{2}{P}'_{3}$. Hence, its $\left(x_{T},y_{T},z_{T}\right)$ coordinates may be expressed as a linear combination of the $\left(x_{1},y_{1},z_{1}\right)$, $\left(x_{2},y_{2},z_{2}\right)$, and $\left(x_{3},y_{3},z_{3}\right)$ coordinates of the points ${P}'_{1}$, ${P}'_{2}$, and ${P}'_{3}$ using barycentric coordinates as indicated by Equation 27.

**Figure 10:**
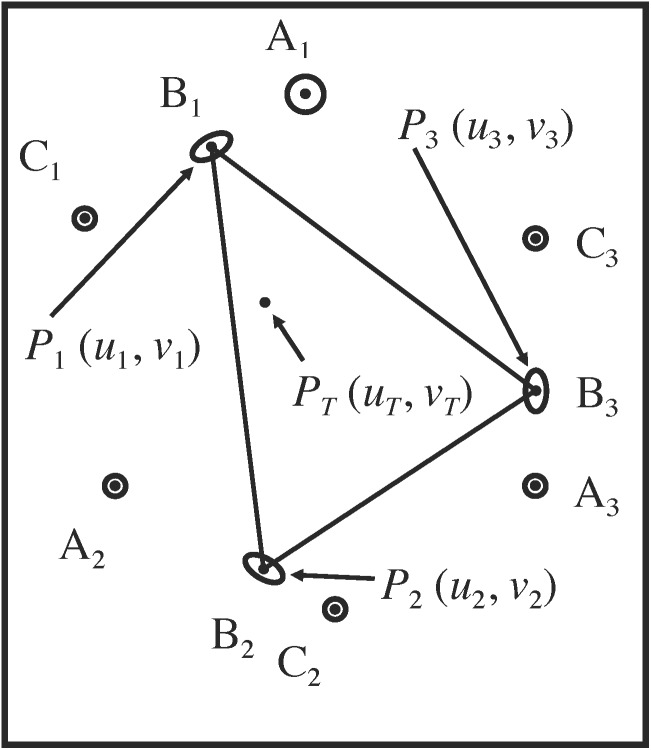
Representation of the Two-Dimensional Coordinate System of the Tomographic Image The cross sections of the three N-localizers create three sets of fiducials $\left \{ \mathrm{A_1,B_1,C_1} \right \}$, $\left \{ \mathrm{A_2,B_2,C_2} \right \}$, and $\left \{ \mathrm{A_3,B_3,C_3} \right \}$ in the tomographic image. Each set contains two circles and one ellipse that are collinear. The centers $P_{1}$, $P_{2}$, and $P_{3}$ of the three ellipses coincide with the respective points of intersection ${P}'_{1}$, ${P}'_{2}$, and ${P}'_{3}$ of the long axes of the three diagonal rods with the tomographic section (Figure 9). The $\left(u_{1},v_{1}\right)$, $\left(u_{2},v_{2}\right)$, and $\left(u_{3},v_{3}\right)$ coordinates of the centers $P_{1}$, $P_{2}$, and $P_{3}$ correspond respectively to the $\left(x_{1},y_{1},z_{1}\right)$, $\left(x_{2},y_{2},z_{2}\right)$, and $\left(x_{3},y_{3},z_{3}\right)$ coordinates of the points of intersection ${P}'_{1}$, ${P}'_{2}$, and ${P}'_{3}$. The target point $P_{T}$ lies in the plane of the triangle $P_{1}P_{2}P_{3}$. Hence, its $\left(u_{T},v_{T}\right)$ coordinates may be expressed as a linear combination of the $\left(u_{1},v_{1}\right)$, $\left(u_{2},v_{2}\right)$, and $\left(u_{3},v_{3}\right)$ coordinates of the points $P_{1}$, $P_{2}$, and $P_{3}$ using barycentric coordinates as indicated by Equation 28.

## Appendices

### Appendix 1: Derivation of Equation 3

Equation 3 transforms $\left(u,v \right )$ coordinates from the two-dimensional coordinate of the tomographic image to the three-dimensional coordinate system of the stereotactic frame to produce $\left(x,y,z \right )$ coordinates. Prior to use in Equation 3, the $\left(u,v \right )$ coordinates are projected onto the $w=1$ plane in three-dimensional space by appending a third coordinate $w=1$ to create $\left(u,v,1 \right )$ coordinates. Equation 3 is derived as follows.

Transformation of coordinates from one three-dimensional coordinate system to another three-dimensional coordinate system may be accomplished via matrix multiplication that operates in a four-dimensional space [9]. However, in order that this four-dimensional space may be used to transform the two-dimensional $\left(u,v \right )$ coordinates into three-dimensional $\left(x,y,z \right )$ coordinates, it is necessary first to create three-dimensional $\left(u,v,0\right )$ coordinates by projecting the $\left(u,v \right )$ coordinates onto the $h=0$ plane in three-dimensional space by appending a third coordinate $h=0$. Then it is necessary to create four-dimensional $\left(u,v,0,1\right )$ coordinates by projecting the $\left(u,v,0\right )$ coordinates onto the $w=1$ hyperplane in four-dimensional space by appending a fourth, homogenous [10] coordinate $w=1$. The $\left(u,v,0,1\right )$ coordinates may be transformed to obtain $\left(x,y,z,1\right )$ coordinates using a four by four transformation matrix that contains the matrix elements $m_{11}$ through $m_{43}$

$$\begin{bmatrix} x_1 & y_1 & z_1 & 1 \\ x_2 & y_2 & z_2 & 1 \\ x_3 & y_3 & z_3 & 1 \end{bmatrix} = \begin{bmatrix} u_1 & v_1 & 0 & 1 \\ u_2 & v_2 & 0 & 1 \\ u_3 & v_3 & 0 & 1 \end{bmatrix} \begin{bmatrix} m_{11} & m_{12} & m_{13} & 0 \\ m_{21} & m_{22} & m_{23} & 0 \\ m_{41} & m_{42} & m_{43} & 0 \\ m_{31} & m_{32} & m_{33} & 1 \end{bmatrix}\;\;\;\;\;\;\;\;\;\;\left(13\right)$$

In Equation 13, the third row of the transformation matrix includes elements $m_{41}$, $m_{42}$, and $m_{43}$ and the fourth row includes elements $m_{31}$, $m_{32}$, and $m_{33}$. This non-standard numbering convention for these matrix elements is convenient to the remainder of this derivation of Equation 3. Also, the matrix elements in the fourth column of this transformation matrix have the values of 0, 0, 0 and 1 because Equation 13 expresses an affine transformation that comprises only scale, rotate and translate operations [9]. These operations accomplish the stretching, rotating, tilting and lifting processes that were described for the membrane analogy in association with Equation 3.

Equation 13 may be rewritten in more compact form as

$$\mathbb{F}=\mathbb{SM}\;\;\;\;\;\;\;\;\;\;\left(14 \right )$$

In Equation 14, $\mathbb{F}$ represents the matrix of $\left(x_{1},y_{1},z_{1},1\right)$, $\left(x_{2},y_{2},z_{2},1\right)$, and $\left(x_{3},y_{3},z_{3},1\right)$ coordinates. $\mathbb{S}$ represents the matrix of $\left(u_{1},v_{1},0,1\right)$, $\left(u_{2},v_{2},0,1\right)$, and $\left(u_{3},v_{3},0,1\right)$ coordinates. $\mathbb{M}$ represents the transformation matrix of elements $m_{11}$ through $m_{43}$ that defines the transformation from the two-dimensional coordinate system of the tomographic image to the three-dimensional coordinate system of the stereotactic frame.

A comparison of Equations 3 and 13 reveals that both equations produce identical results for the $\left(x_{1},y_{1},z_{1}\right)$, $\left(x_{2},y_{2},z_{2}\right)$, and $\left(x_{3},y_{3},z_{3}\right)$ coordinates. The third column of $\mathbb{S}$ and the third row of $\mathbb{M}$ do not affect the $\left(x_{1},y_{1},z_{1}\right)$, $\left(x_{2},y_{2},z_{2}\right)$, and $\left(x_{3},y_{3},z_{3}\right)$ coordinates. The fourth column of $\mathbb{M}$ affects only the fourth column of $\mathbb{F}$ but does not affect the $\left(x_{1},y_{1},z_{1}\right)$, $\left(x_{2},y_{2},z_{2}\right)$, and $\left(x_{3},y_{3},z_{3}\right)$ coordinates. Hence, these columns and this row may be removed from $\mathbb{F}$, $\mathbb{S}$, and $\mathbb{M}$ without affecting the result of Equation 13. Their removal yields Equation 3, thus completing the derivation of Equation 3.

There is a significant difference between Equations 3 and 13. None of the matrices in Equation 13 have an inverse because neither $\mathbb{F}$ nor $\mathbb{S}$ is a square matrix. In contrast, the matrices $\mathrm{\textbf{F}}$, $\mathrm{\textbf{S}}$, and $\mathrm{\textbf{M}}$ in Equation 3 potentially have inverses because these matrices are square matrices. Equations 5, 7, and 8 require that these matrices have inverses. Hence, in order to express the transformation from the two-dimensional coordinate system of the tomographic image to the three-dimensional coordinate system of the stereotactic frame and vice versa, Equation 3 must be used instead of Equation 13.

### Appendix 2: Derivation of the Distance \begin{document}w-1\end{document}

Equation 9 calculates the interpolant or extrapolant $t$ in the $\left(u,v,w\right)$ coordinate system of the tomographic image. This interpolant or extrapolant is calculated in terms of the perpendicular distance $w-1$ from a point $P$ to the plane of the tomographic image. The distance $w-1$ is derived as follows.

In the three-dimensional $\left(x,y,z\right)$ coordinate system of the stereotactic frame, the equation for the central plane of the tomographic section is given by the following equation that involves a determinant [11-12]

$$\begin{vmatrix} x & y & z & 1 \\ x_1 & y_1 & z_1 &1 \\ x_2 & y_2 & z_2 & 1 \\ x_3 & y_3 & z_3 & 1 \end{vmatrix} = 0\;\;\;\;\;\;\;\;\;\;\left(15 \right )$$

Expanding this determinant using the cofactors [12] of the elements $x$, $y$, $z$, and 1 in the first row of the determinant yields

$$x\:\begin{vmatrix} y_1 & z_1 & 1\\ y_2 & z_2 & 1\\ y_3 & z_3 & 1 \end{vmatrix} - y\:\begin{vmatrix} x_1 & z_1 & 1\\ x_2 & z_2 & 1\\ x_3 & z_3 & 1 \end{vmatrix} + z\:\begin{vmatrix} x_1 & y_1 & 1\\ x_2 & y_2 & 1\\ x_3 & y_3 & 1 \end{vmatrix} - \begin{vmatrix} x_1 & y_1 & z_1\\ x_2 & y_2 & z_2\\ x_3 & y_3 & z_3 \end{vmatrix} = 0\;\;\;\;\;\;\;\;\;\;\left(16 \right)$$

Equation 16 may be rewritten in more compact form as

$$Ax + By+Cz+D = 0\;\;\;\;\;\;\;\;\;\;\left(17 \right )$$

where $A$, $B$, $C$, and $D$ represent the determinants in Equation 16. The determinants $A$, $B$, and $C$ may be expanded using the cofactors of the elements in their third columns as follows

$$\begin{matrix} A= \begin{vmatrix} y_1 & z_1 & 1\\ y_2 & z_2 & 1\\ y_3 & z_3 & 1 \end{vmatrix}= \begin{vmatrix} y_2 & z_2\\ y_3 & z_3 \end{vmatrix}-\begin{vmatrix} y_1 & z_1\\ y_3 & z_3 \end{vmatrix}+\begin{vmatrix} y_1 & z_1\\ y_2 & z_2 \end{vmatrix} & \left(18a \right ) \\ B= -\begin{vmatrix} x_1 & z_1 & 1\\ x_2 & z_2 & 1\\ x_3 & z_3 & 1 \end{vmatrix} = -\begin{vmatrix} x_2 & z_2\\ x_3 & z_3 \end{vmatrix}+\begin{vmatrix} x_1 & z_1\\ x_3 & z_3 \end{vmatrix}-\begin{vmatrix} x_1 & z_1\\ x_2 & z_2 \end{vmatrix} & \left(18b \right ) \\ C= \begin{vmatrix} x_1 & y_1 & 1\\ x_2 & y_2 & 1\\ x_3 & y_3 & 1 \end{vmatrix} = \begin{vmatrix} x_2 & y_2\\ x_3 & y_3 \end{vmatrix}-\begin{vmatrix} x_1 & y_1\\ x_3 & y_3 \end{vmatrix}+\begin{vmatrix} x_1 & y_1\\ x_2 & y_2 \end{vmatrix} & \left(18c \right ) \\ D= - \begin{vmatrix} x_1 & y_1 & z_1\\ x_2 & y_2 & z_2\\ x_3 & y_3 & z_3 \end{vmatrix} & \left(18d \right ) \end{matrix}$$

The normalized perpendicular distance $d$ from a point ${P}'$, which has coordinates $\left(x,y,z\right)$, to the central plane of the tomographic section may be calculated as [11]

$$d=\frac{Ax+By+Cz+D}{\sqrt{A^2+B^2+C^2}}\;\;\;\;\;\;\;\;\;\;\left(19 \right )$$

This equation for the normalized perpendicular distance will be compared to the equation for the distance $w-1$ that is derived below.

In order to calculate the distance $w-1$, the $\left(u,v,w\right)$ coordinates of the point $P$ that corresponds to the point ${P}'$ are obtained by transforming the $\left(x,y,z\right)$ coordinates of the point ${P}'$ via Equation 8 then by substituting the definitions of matrices $\mathrm{\textbf{F}}$, $\mathrm{\textbf{S}}$, and $\mathrm{\textbf{M}}$ from Equation 3

$$\left[u\;v\;w\right] = \left[x\;y\;z \right ] \mathrm{\textbf{M}}^{-1} = \left[x\;y\;z \right ] \mathrm{\textbf{F}}^{-1} \mathrm{\textbf{S}} = \left[x\;y\;z \right ] \begin{bmatrix} x_1 & y_1 & z_1\\ x_2 & y_2 & z_2\\ x_3 & y_3 & z_3 \end{bmatrix}^{-1} \begin{bmatrix} u_1 & v_1 & 1 \\ u_2 & v_2 & 1 \\ u_3 & v_3 & 1 \end{bmatrix}\;\;\;\;\;\left(20 \right )$$

Substituting the inverse of the matrix $\mathrm{\textbf{F}}$, which is defined as its adjoint [12] divided by its determinant, into Equation 20 yields

$$\left[u\;v\;w \right ] = \left[x\;y\;z \right ] \frac{ \begin{bmatrix} \begin{vmatrix} y_2 & z_2\\ y_3 & z_3 \end{vmatrix} & -\begin{vmatrix} y_1 & z_1\\ y_3 & z_3 \end{vmatrix} & \begin{vmatrix} y_1 & z_1\\ y_2 & z_2 \end{vmatrix} \\ -\begin{vmatrix} x_2 & z_2\\ x_3 & z_3 \end{vmatrix} & \begin{vmatrix} x_1 & z_1\\ x_3 & z_3 \end{vmatrix} & -\begin{vmatrix} x_1 & z_1\\ x_2 & z_2 \end{vmatrix} \\ \begin{vmatrix} x_2 & y_2\\ x_3 & y_3 \end{vmatrix} & -\begin{vmatrix} x_1 & y_1\\ x_3 & y_3 \end{vmatrix} & \begin{vmatrix} x_1 & y_1\\ x_2 & y_2 \end{vmatrix} \end{bmatrix}}{ \begin{vmatrix} x_1 & y_1 & z_1\\ x_2 & y_2 & z_2\\ x_3 & y_3 & z_3 \end{vmatrix} } \begin{bmatrix} u_1 & v_1 & 1 \\ u_2 & v_2 & 1 \\ u_3 & v_3 & 1 \end{bmatrix}\;\;\;\;\;\left(21 \right)$$

Transformation of the $\left(x,y,z\right)$ coordinates of the point ${P}'$ to obtain only the $w$-coordinate of the point $P$ requires only the vector from the third column of $\mathrm{\textbf{M}}^{-1}$. Hence, keeping only the third column of the matrix that results from the post-multiplication of $\mathrm{\textbf{F}}^{-1}$ by $\mathrm{\textbf{S}}$ produces the following expression for $w$ that contains a three-element column vector

$$w = \left[x\;y\;z \right ] \frac{ \begin{bmatrix} \begin{bmatrix} y_2 & z_2\\ y_3 & z_3 \end{bmatrix} -\begin{vmatrix} y_1 & z_1\\ y_3 & z_3 \end{vmatrix} + \begin{vmatrix} y_1 & z_1\\ y_2 & z_2 \end{vmatrix} \\ -\begin{vmatrix} x_2 & z_2\\ x_3 & z_3 \end{vmatrix} + \begin{vmatrix} x_1 & z_1\\ x_3 & z_3 \end{vmatrix} -\begin{vmatrix} x_1 & z_1\\ x_2 & z_2 \end{vmatrix} \\ \begin{vmatrix} x_2 & y_2\\ x_3 & y_3 \end{vmatrix} -\begin{vmatrix} x_1 & y_1\\ x_3 & y_3 \end{vmatrix} + \begin{vmatrix} x_1 & y_1\\ x_2 & y_2 \end{vmatrix} \end{bmatrix} }{ \begin{vmatrix} x_1 & y_1 & z_1\\ x_2 & y_2 & z_2\\ x_3 & y_3 & z_3 \end{vmatrix} }\;\;\;\;\;\;\;\;\;\;\left(22 \right)$$

Rewriting Equation 22 in more compact form using the definitions of $A$, $B$, $C$, and $D$ from Equation 18 yields

$$w = \left[ x\;y\;z \right ] \frac{ \begin{bmatrix} A \\ B \\ C \end{bmatrix} } { -D }\;\;\;\;\;\;\;\;\;\;\left(23 \right )$$

Performing the vector multiplication of Equation 23 produces the $w$-coordinate of the point $P$

$$w = \frac{Ax + By + Cz}{-D}\;\;\;\;\;\;\;\;\;\;\left(24 \right )$$

The perpendicular distance from the point $P$ to the plane of the tomographic image is given by $w-1$

$$w-1 = \frac{Ax + By + Cz + D}{-D}\;\;\;\;\;\;\;\;\;\;\left(25 \right )$$

Comparison of Equation 25 to Equation 19 reveals that the numerators of these equations are identical but their denominators differ, as can be demonstrated by expanding the determinants $A$, $B$, $C$, and $D$ then showing that $D^2 \neq A^2 + B^2 + C^2$. Thus, the distance that is calculated using Equation 25 differs by a factor of $-\sqrt{A^2 + B^2 + C^2} / D$ from the normalized distance that is calculated using Equation 19. However, this factor is not relevant to the interpolant or extrapolant $t$ that is calculated via Equation 9 because Equation 9 calculates a ratio of distances that eliminates this factor. Hence, $w-1$ may be used to construct the interpolant or extrapolant $t$ according to Equation 9.

### Appendix 3: Transformation of the Target Point \begin{document}P_T\end{document} and the Analogous Target Point \begin{document}{P}'_T\end{document}

Equation 3 expresses the transformation of points $P_{1}$, $P_{2}$, and $P_{3}$ from the two-dimensional coordinate system of the tomographic section to obtain the analogous points ${P}'_{1}$, ${P}'_{2}$, and ${P}'_{3}$ in the three-dimensional coordinate system of the stereotactic frame. However, the above discussion of the membrane analogy asserts that any point that lies in the plane of points $P_{1}$, $P_{2}$, and $P_{3}$ may be transformed from the two-dimensional coordinate system of the tomographic section to the three-dimensional coordinate system of the stereotactic frame via the same transformation that transforms points $P_{1}$, $P_{2}$, and $P_{3}$. This assertion obtains due to the principles of linear algebra and is proved as follows.

Equation 3 transforms *en masse* the $\left(u_{1},v_{1},1\right)$, $\left(u_{2},v_{2},1\right)$, and $\left(u_{3},v_{3},1\right)$ coordinates of the three points $P_{1}$, $P_{2}$, and $P_{3}$ to create the $\left(x_{1},y_{1},z_{1}\right)$, $\left(x_{2},y_{2},z_{2}\right)$, and $\left(x_{3},y_{3},z_{3}\right)$ coordinates of the three points ${P}'_{1}$, ${P}'_{2}$, and ${P}'_{3}$. An alternative is to transform separately the $\left(u_{1},v_{1},1\right)$, $\left(u_{2},v_{2},1\right)$, and $\left(u_{3},v_{3},1\right)$ coordinates of the three points $P_{1}$, $P_{2}$, and $P_{3}$ to obtain the $\left(x_{1},y_{1},z_{1}\right)$, $\left(x_{2},y_{2},z_{2}\right)$, and $\left(x_{3},y_{3},z_{3}\right)$ coordinates of the three points ${P}'_{1}$, ${P}'_{2}$, and ${P}'_{3}$, respectively

$${P}'_1 = \begin{bmatrix}x_1\;y_1\;z_1\end{bmatrix} = \begin{bmatrix}u_1\;v_1\;1\end{bmatrix} \begin{bmatrix} m_{11} & m_{12} & m_{13} \\ m_{21} & m_{22} & m_{23} \\ m_{31} & m_{32} & m_{33} \end{bmatrix} = P_1\mathbf{M} \;\;\;\;\;\;\;\;\;\; \left(26a\right)$$

$${P}'_2 = \begin{bmatrix}x_2\;y_2\;z_2\end{bmatrix} = \begin{bmatrix}u_2\;v_2\;1\end{bmatrix} \begin{bmatrix} m_{11} & m_{12} & m_{13} \\ m_{21} & m_{22} & m_{23} \\ m_{31} & m_{32} & m_{33} \end{bmatrix} = P_2\mathbf{M} \;\;\;\;\;\;\;\;\;\; \left(26b\right)$$

$${P}'_3 = \begin{bmatrix}x_3\;y_3\;z_3\end{bmatrix} = \begin{bmatrix}u_3\;v_3\;1\end{bmatrix} \begin{bmatrix} m_{11} & m_{12} & m_{13} \\ m_{21} & m_{22} & m_{23} \\ m_{31} & m_{32} & m_{33} \end{bmatrix} = P_3\mathbf{M} \;\;\;\;\;\;\;\;\;\; \left(26c\right)$$

Equation 26 produces the same result for the $\left(x_{1},y_{1},z_{1}\right)$, $\left(x_{2},y_{2},z_{2}\right)$, and $\left(x_{3},y_{3},z_{3}\right)$ coordinates of points ${P}'_{1}$, ${P}'_{2}$, and ${P}'_{3}$ as does Equation 3.

It is possible to represent any point that lies in a plane defined by three other points as a linear combination of those three points using the barycentric coordinates $b_1$, $b_2$, and $b_3$ that satisfy the condition $b_1 + b_2 + b_3 = 1$ [13]. For example, with reference to Figure 9, the target point ${P}'_T$ may be represented as a linear combination of the three points ${P}'_{1}$, ${P}'_{2}$, and ${P}'_{3}$

$${P}'_T = b_1{P}'_1 + b_2{P}'_2 + b_3{P}'_3 \;\;\;\;\;\;\;\;\;\; \left(27\right)$$

Similarly, with reference to Figure 10, the analogous target point $P_T$ may be represented as a linear combination of the three points $P_1$, $P_2$, and $P_3$.

$$P_T = b_1P_1 + b_2P_2 + b_3P_3 \;\;\;\;\;\;\;\;\;\; \left(28\right)$$

Because the matrix $\mathbf{M}$ that transforms $\mathbf{S}$ into $\mathbf{F}$ via Equation 4 describes a linear transformation, the barycentric coordinates $b_1$, $b_2$, and $b_3$ apply to both $P_T$ and ${P}'_T$. Hence, these barycentric coordinates may be used in both Equation 27 and Equation 28. These equations describe interpolation in a plane that is analogous to the interpolation along a line that is expressed by Equation 1.

Using the matrix $\mathbf{M}$ to transform the point $P_T$ as shown in Equation 6 and substituting Equations 26-28 yields

\begin{align*}P_T\:\mathbf{M} & = \left(b_1P_1 + b_2P_2 + b_3P_3\right)\mathbf{M} \\ & = b_1P_1\mathbf{M} + b_2P_2\mathbf{M} + b_3P_3\mathbf{M} \\ & = b_1{P}'_1 + b_2{P}'_2 + b_3 {P}'_3 \\ & = {P}'_T\end{align*} \;\;\;\;\;\;\;\;\;\; \left(29\right)

Eliminating the intermediate steps from Equation 29 and showing explicitly the $\left(x_T,y_T,z_T\right)$ coordinates of ${P}'_T$ and the $\left(u_T,v_T\right)$ coordinates of $P_T$ yields 

$${P}'_T = \begin{bmatrix}x_T\;y_T\;z_T\end{bmatrix} = \begin{bmatrix}u_T\;v_T\;1\end{bmatrix}\mathbf{M} = P_T\:\mathbf{M} \;\;\;\;\;\;\;\;\;\; \left(30\right)$$

Equation 30 proves that any point $P_T$ that lies in the tomographic image may be transformed from the two-dimensional coordinate system of that image to the three-dimensional coordinate system of the stereotactic frame to obtain the analogous point ${P}'_T$.

Using the inverse matrix $\mathbf{M}^{-1}$ to transform the point ${P}'_T$ as shown in Equation 8 and substituting Equations 26-28 yields

\begin{align*}{P}'_T\,\mathbf{M}^{-1} & = \left(b_1{P}'_1 + b_2{P}'_2 + b_3 {P}'_3\right)\mathbf{M}^{-1} \\ & = b_1{P}'_1\,\mathbf{M}^{-1} + b_2{P}'_2\,\mathbf{M}^{-1} + b_3 {P}'_3\,\mathbf{M}^{-1} \\ & = b_1P_1 + b_2P_2 + b_3P_3 \\ & = P_T\end{align*} \;\;\;\;\;\;\;\;\;\; \left(31\right)

Eliminating the intermediate steps from Equation 31 and showing explicitly the $\left(x_T,y_T,z_T\right)$ coordinates of ${P}'_T$ and the $\left(u_T,v_T\right)$ coordinates of $P_T$ yields

$$P_T = \begin{bmatrix}u_T\;v_T\;1\end{bmatrix} = \begin{bmatrix}x_T\;y_T\;z_T\end{bmatrix}\mathbf{M}^{-1} = {P}'_T\:\mathbf{M}^{-1} \;\;\;\;\;\;\;\;\;\; \left(32\right)$$

Equation 32 proves that any point ${P}'_T$ that lies in the plane of the tomographic section may be transformed from the three-dimensional coordinate system of the stereotactic frame to the two-dimensional coordinate system of the tomographic image to obtain the analogous point $P_T$.
